# Meta‐analysis of peripheral insulin‐like growth factor 1 levels in schizophrenia

**DOI:** 10.1002/brb3.2819

**Published:** 2022-11-30

**Authors:** Ana V. Pejcic, Slobodan M. Jankovic, Vladimir Janjic, Milan Djordjic, Jovana Z. Milosavljevic, Milos N. Milosavljevic

**Affiliations:** ^1^ Department of Pharmacology and Toxicology, Faculty of Medical Sciences University of Kragujevac Kragujevac Serbia; ^2^ Department of Psychiatry, Faculty of Medical Sciences University of Kragujevac Kragujevac Serbia; ^3^ Department of Communication Skills, Ethics and Psychology, Faculty of Medical Sciences University of Kragujevac Kragujevac Serbia; ^4^ Department of Anatomy, Faculty of Medical Sciences University of Kragujevac Kragujevac Serbia

**Keywords:** insulin‐like growth factor 1, meta‐analysis, schizophrenia

## Abstract

**Objective:**

We aimed to investigate if there is a significant difference in peripheral insulin‐like growth factor 1 (IGF‐1) levels between schizophrenia patients and healthy controls and to determine whether a difference exists before and after initiation of antipsychotics.

**Methods:**

PubMed/MEDLINE, Scopus, and Web of Science were searched up to March 27, 2022. Original clinical studies of any type that reported peripheral blood, serum or plasma IGF‐1 levels measured after fasting in schizophrenia patients and/or healthy control group were selected based on inclusion and exclusion criteria. Data were analyzed using Meta‐Essentials: Workbooks for meta‐analysis and pooled through random‐effects meta‐analyses.

**Results:**

Twelve publications met eligibility criteria. Schizophrenia patients under antipsychotic treatment had significantly lower peripheral IGF‐1 levels compared to healthy controls (*n* = 632, Hedges’ g –0.42, 95% CI from –0.79 to –0.04, *p* = .006, I^2^ = 70.38%), while no significant difference was found between schizophrenia patients regardless of the antipsychotic treatment status and healthy controls, as well as between antipsychotic naïve or free schizophrenia patients and healthy controls, and before and after initiation of antipsychotic treatment. However, high heterogeneity was observed and its potential sources in some of the subgroup analyses included sample type and region.

**Conclusions:**

Schizophrenia patients under antipsychotic treatment seem to have lower peripheral IGF‐1 levels compared to healthy controls. Additional studies with larger and more homogenous samples are needed to confirm these findings.

## Graphical abstract

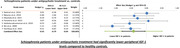

## 1 INTRODUCTION

Schizophrenia is a severe psychiatric disorder characterized by three categories of symptoms: positive (hallucinations, delusions, thought disorders), negative (avolition, social withdrawal, anhedonia, poverty of thought), and cognitive (impairment of executive functions, memory and speed of mental processing) (Marder & Cannon, 2019; Okamoto et al., 2021). It is a significant health issue that affects approximately 1% of the population and has substantial social and economic consequences, as patients are frequently unemployed and homeless (Charlson et al., 2018; Yesilkaya et al., 2021). Its cause appears to lie in a disruption in brain development caused by genetic and/or environmental factors (Owen et al., 2016), but due to its relative complexity and heterogeneity, the etiology and exact pathophysiological mechanisms are not yet completely understood (Hany et al., 2022). Although dopamine dysfunction has long been linked to schizophrenia, accumulating evidence indicates that there are additional abnormalities in other neurotransmitters such as glutamate, serotonin, and gamma‐aminobutyric acid (Yang & Tsai, 2017). There are indications that the insulin‐like growth factor 1 (IGF‐1) pathway may also be involved in the pathophysiology of schizophrenia but its precise role has not yet been determined (Okamoto et al., 2021).

IGF‐1, also called somatomedin C, is a peptide of 70 amino acids that is produced primarily by the liver (Kasprzak, 2021). The level of growth hormone in the blood controls its synthesis and secretion (Chen et al., 2020). IGF‐1 is functional in all tissues and affects overall growth and development (Palomino et al., 2013). It also crosses the blood–brain barrier and is involved in neurogenesis, synaptogenesis, myelination, and dendritic branching during brain development (Gunnell et al., 2007). IGF‐1 signals via Akt/protein kinase B, which can increase myelination in central nervous system neurons by improving the functions of oligodendrocytes and preventing glutamate‐induced apoptosis (Ness et al., 2002; Yesilkaya et al., 2021). So far, several studies comparing peripheral IGF‐1 levels between schizophrenia patients and healthy controls have been conducted. In some of them, peripheral IGF‐1 levels were reported to be lower in schizophrenia patients (Chao et al., 2020; Venkatasubramanian et al., 2007; Wu et al., 2008), while other studies either found higher levels (Chen et al., 2021) or no difference (Demirel et al., 2014; Okamoto et al., 2021; Teja, 2018) compared to healthy controls. These inconsistencies could be a result of differences in sample sizes or specific characteristics of the schizophrenia groups, such as disease and antipsychotic treatment status.

Therefore, the aim of our meta‐analysis was to investigate if there is a significant difference in peripheral IGF‐1 levels between schizophrenia patients and healthy controls, as well as to determine whether a difference in peripheral IGF‐1 levels exists before and after initiation of antipsychotic treatment.

## 2 MATERIALS AND METHODS

This meta‐analysis was registered in the international prospective register of systematic reviews under the registration number CRD42022320394, and its reporting was guided by the standards of the meta‐analysis of observational studies in epidemiology guidelines (Stroup et al., 2000) and preferred reporting items for systematic review and meta‐analysis (PRISMA) statement (Page et al., 2021).

### 2.1 Eligibility criteria

Inclusion criteria were as follows: (1) an original clinical study of any type that reported peripheral blood, serum, or plasma IGF‐1 levels measured after a fasting period in patients with schizophrenia and/or healthy (normal) control group; (2) patients were diagnosed with schizophrenia according to the diagnostic and statistical manual of mental disorders (DSM) or international statistical classification of diseases and related health problems (ICD) criteria; (3) peripheral IGF‐1 levels in patients with schizophrenia were compared with healthy (normal) controls and/or before and after initiation of antipsychotic treatment. Exclusion criteria were as follows: (1) studies reporting peripheral IGF‐1 levels in which schizophrenia patients were combined with patients with some other psychiatric diagnosis; (2) mean or standard deviation (SD) of peripheral IGF‐1 levels could not be extracted, calculated, or obtained or effect size could not be calculated from other reported values (e.g. *t* value, *F* value); (3) studies with unavailable full text and conference abstracts in which relevant data are not available in the text of the abstract or could not be obtained from the authors upon request.

### 2.2 Search strategy

Three electronic databases were searched independently by two authors (AVP, MNM) from the beginning of indexing up to March 27, 2022, without any language or date restriction: PubMed/MEDLINE, Scopus, and Web of Science. A complete search strategy for each database is shown in Table 1. Both backward and forward citation searching on publications that met the eligibility criteria of the meta‐analysis was performed. Backward citation searching was performed by inspecting the references that were cited in these studies, while forward citation searching was performed by using Google Scholar citation index on March 28, 2022, to identify studies that cited these studies. Initially, two authors (AVP, MNM) independently reviewed the eligibility of retrieved publications based on the title and abstract. When the title and information supplied in the abstract were insufficient to determine whether the publication properly corresponded to the research topic, the full text of the publication was retrieved and evaluated. We tried to contact the authors of six publications (Akanji et al., 2007; Chen et al., 2021; Huizer et al., 2006, 2007; Lee & Kim, 2007; Ntouros et al., 2018) with a request to provide information about relevant data that were not available in the retrieved publications. Publications were included in the meta‐analysis if all authors agreed that the eligibility criteria were met. Disagreements between individual judgments were resolved by the senior author (SMJ).

**TABLE 1:** Detailed search strategy for databases

Database	Search strategy
PubMed/MEDLINE	(“insulin like growth factor i”[MeSH Terms] OR “insulin like growth factor i”[All Fields] OR “igf 1″[All Fields] OR (“insulin like growth factor i”[MeSH Terms] OR “insulin like growth factor i”[All Fields] OR “insulin like growth factor 1″[All Fields]) OR “somatomedin C”[All Fields]) AND (“schizophrenia”[MeSH Terms] OR “schizophrenia”[All Fields] OR “schizophrenias”[All Fields] OR “schizophrenia s”[All Fields])
Web of Science	In all databases and all collections (Web of Science Core Collection, KCI‐Korean Journal Database, Russian Science Citation Index, SciELO Citation Index): TS = (IGF‐1 OR (insulin‐like growth factor 1) OR (“somatomedin C”)) AND TS = schizophrenia
Scopus	TITLE‐ABS ((igf‐1 OR (insulin‐like AND growth AND factor 1) OR (“somatomedin C”)) AND schizophrenia)

### 2.3 Data extraction

The data extraction sheet was created and two authors (AVP, MNM) independently extracted the following data from the included publications: study ID, citation, country/region, sample characteristics (e.g., participant groups, sample sizes, age, number of males/females, information regarding diagnosis of schizophrenia and antipsychotic treatment, main characteristics of groups, body mass index), mean and SD of the peripheral IGF‐1 levels or if not available alternative values for calculation of effect size (e.g., *t* value), method of peripheral IGF‐1 measurement, blood sample type (peripheral blood, serum, plasma), and study conclusions. Another author (JZM) created the final extraction table after collating the two tables. The conversion factor of 7.65 was used for the conversion of peripheral IGF‐1 levels reported in nmol/L to ng/ml (1 ng/ml = 1 μg/L) (Andersen et al., 2015; Yovich et al., 2020). When reported as such, standard errors were converted to SDs using the number of patients.

### 2.4 Methodological quality (risk of bias) assessment

The methodological index for non‐randomized studies (MINORS) tool (Slim et al., 2003) was used to assess the methodological quality (risk of bias) of the included studies. The MINORS tool has a total of 12 methodological items: the first eight items are applicable to both non‐comparative and comparative studies, while the last four items are applicable only to studies involving two or more groups (Slim et al., 2003). Each item is scored from 0 to 2: 0 (not reported), 1 (reported but inadequate), and 2 (reported and adequate) (Slim et al., 2003). For non‐comparative studies, the global ideal score is 16, while for comparative studies, it is 24 (Slim et al., 2003).

### 2.5 Data analysis

Meta‐Essentials: Workbooks for meta‐analysis (Version 1.5) (Suurmond et al., 2017) was used to analyze the data. To combine the results from the studies in meta‐analyses, random effects model was used, while combined effects sizes were estimated using Hedges’ g with its 95% confidence interval (CI), prediction interval, and corresponding tests of significance. Hedges' g was chosen as an appropriate statistic for the combined effect size because it tends to produce less bias in small samples. The combined effect size was considered significant only if both the associated 95% CI did not include zero and the associated two‐tailed *p* value was significant (i.e., < .05).

Values of means and SDs of peripheral IGF‐1 levels or other available information (e.g., *t* value) and a corresponding number of schizophrenia patients and healthy controls were entered into Workbook 3 “Differences between independent groups—continuous data.xlsx.” Three main comparisons were performed using this workbook: (1) schizophrenia patients regardless of the antipsychotic treatment status versus healthy (normal) controls, (2) antipsychotic naïve or free schizophrenia patients versus healthy (normal) controls, and (3) schizophrenia patients under antipsychotic treatment versus healthy (normal) controls.

Values of means and SDs of peripheral IGF‐1 levels before (baseline) and after initiation of antipsychotic treatment and a corresponding number of patients (baseline if there was a loss to follow up) were entered into Workbook 4 “Differences between dependent groups—continuous data.xlsx.” Because none of the meta‐analyzed studies provided a correlation coefficient (*r*) describing the relationship between pairs of observations, all studies were assigned the same *r* value and the analysis was repeated with different correlation coefficients: .25 (poor), .60 (moderate), and .85 (very strong) (Johansson et al., 2020; Van Rhee et al., 2015).

In case of studies of long duration, in which results were presented for several periods, we selected first available (i.e., baseline) values in the first analysis involving schizophrenia patients regardless of the antipsychotic treatment status because in this way the largest number of patients would be included in the analysis. For the remaining analyses, we selected the longest follow‐up after initiation of antipsychotic treatment from each study. When studies reported data for two subgroups of schizophrenia patients, we calculated pooled means and SDs and used them in the meta‐analysis, to ensure that the results were not influenced by one study contributing a disproportionate number of data points to the analysis and to avoid over‐sampling the control groups, as described in *Cochrane Handbook for Systematic Reviews of Interventions* (Higgins et al., 2022).

Sensitivity analysis was carried out by excluding individual studies one at a time and recalculating the combined effect size estimates for the remaining studies.

Cochran's Q test and I^2^ statistic were used to evaluate heterogeneity among the studies. Cochran's Q test informs us whether heterogeneity is present or not. Because Cochran's Q test can have low power when studies have a small sample size or are few in number, a *p* value of < .10 was considered to indicate the presence of statistically significant heterogeneity (rather than the conventional level of .05) (Higgins et al., 2022; West et al., 2010). I^2^ is an estimate of the proportion of total variation across studies that can be attributed to true heterogeneity rather than chance (0% indicates no observed heterogeneity, while larger values indicate increasing heterogeneity). An I^2^ value > 30% was considered significant.

To explore sources of significant heterogeneity additional subgroup analyses were performed in relation to sample type (serum, plasma), measurement method (ELISA, other), and region (Asia, Europe), along with the moderator analysis with available continuous moderator variables including schizophrenia patients’ mean age, body mass index (BMI), gender ratio (percentage of male participants), and duration of antipsychotic treatment (only for comparison of levels before and after initiation of antipsychotic treatment).

Three methods were used to evaluate publication bias: funnel plot with trim‐and‐fill analysis (Duval & Tweedie, 2000a, 2000b), Egger regression test (Egger et al., 1997), and Begg and Mazumdar rank correlation test (Begg & Mazumdar, 1994). A *p* value < .05 indicated the presence of significant publication bias in the Egger regression test and Begg and Mazumdar rank correlation test.

## 3 Results

### 3.1 Results of the search and selection process

**FIGURE 1:**
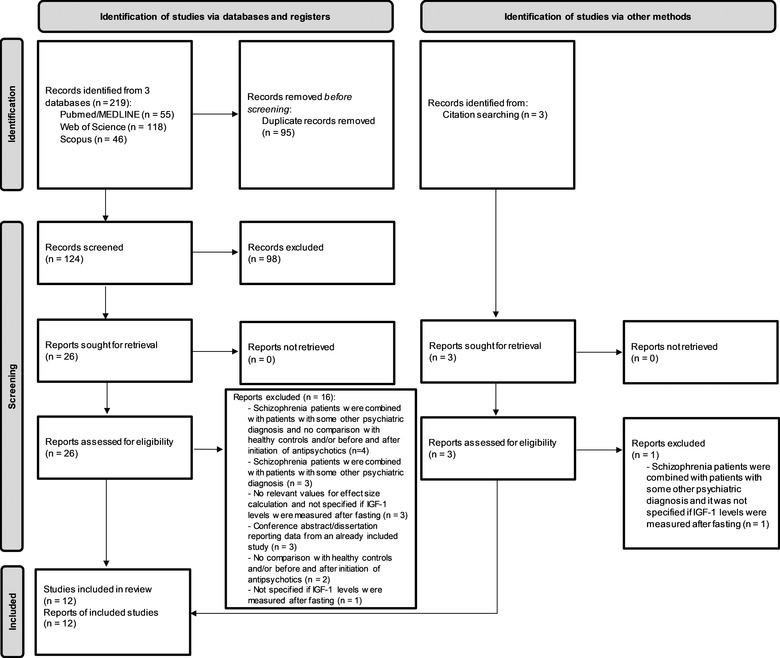
Results of the search and selection process (PRISMA flow diagram)

PRISMA flow diagram describing the results of the search and selection process is provided in Figure 1. Authors of two publications replied: one provided requested data, so this publication was included in the analysis (Ntouros et al., 2018), while the other one was not able to provide answers due to the loss of the data, so this publication was excluded (Lee & Kim, 2007). Authors of other publications did not respond, so three of these were also excluded (Akanji et al., 2007; Huizer et al., 2006, 2007), while one (Chen et al., 2021) was included only in the meta‐analyses for which the data were adequately reported in the published article. A total of 12 studies met all eligibility criteria (Chao et al., 2020; Chen et al., 2021; Demirel et al., 2014; Howes et al., 2004; Ntouros et al., 2018; Okamoto et al., 2021; Palomino et al., 2013; Teja, 2018; Venkatasubramanian et al., 2007, 2010; Wu et al., 2008; Yesilkaya et al., 2021). The list of excluded publications with reasons for exclusion is provided in Supporting Information.

### 3.2 Study characteristics

Characteristics of included studies are summarized in Table 2. Studies were conducted in India (*n* = 3), China (*n* = 2), Turkey (*n* = 2), Japan (*n* = 1), Spain (*n* = 1), Taiwan (*n* = 1), Greece (*n* = 1), and the United Kingdom (*n* = 1). The most frequently used sample type and measurement method were serum (*n* = 7) and enzyme‐linked immunosorbent assay (*n* = 6), respectively.

**TABLE 2:** Characteristics of included studies

				SCH group			Healthy (normal) control group			
	Study	Country or region	Blood sample type, measurement method	Diagnostic criteria, main characteristics and AP treatment status	*n* (gender: M/F), mean age ± SD in years, mean BMI ± SD (kg/m^2^)	Mean ± SD of peripheral IGF‐1 levels (in ng/ml)	Main characteristics	*n* (gender: M/F), mean age ± SD in years, mean BMI ± SD (kg/m^2^)	Mean ± SD of peripheral IGF‐1 levels in (in ng/ml)	Conclusion
1	Chao et al. (2020)	China	Serum, ELISA	DSM‐IV, not taken AP for at least 3 months prior to taking part in the study.	30 (16/14), 30.47 ± 8.53, 21.70 ± 2.23	114.96 ± 65.85	Healthy controls matched by gender, age, education, and BMI.	26 (12/14), 34.38 ± 9.98, 20.86 ± 1.96	183.43 ± 86.42	Peripheral IGF‐1 levels significantly lower in SCH patients (p = .001).
2	Chen et al. (2021)	China	Plasma, ELISA	DSM‐IV, duration of symptoms ≤5 years, never taken AP or if previously treated, the usage was < 2 weeks for a total lifetime. After baseline evaluation, all were given risperidone.	Baseline: 113 (45/68) After 10 weeks: 89 *Other information not adequately reported.	Baseline: 214.45 ± 33.42 10 weeks: 202.29 ± 32.40	Healthy controls similar in age, gender, education, smoking status, and waist–hip ratio.	58 *Other information not adequately reported.	*Other information not adequately reported.	Baseline peripheral IGF‐1 levels in SCH patients significantly higher than in controls (*t* = 2.42, *p* = .017). After 10 weeks peripheral IGF‐1 levels in patients decreased significantly (*p* < .01).
3	Demirel et al. (2014)	Turkey	Plasma, RIA	DSM‐IV, chronic SCH, taking atypical AP either as monotherapy or as a part of a combination therapy.	50 (37/13), 36.46 ± 11.20, 29.77 ± 5.38	176.06 ± 81.65	Control group had not been diagnosed as having any axis 1 disorder according to the SCID‐I.	50 (32/18), 35.54 ± 9.20, 25.74 ± 4.56	175.04 ± 64.01	Mean peripheral IGF‐1 levels did not differ between groups (*p* = 1.000).
4	Howes et al. (2004)	The United Kingdom	Blood, automated chemiluminescence immunoassay	DSM‐IV, SCH patients who commenced clozapine treatment.	19 (9/10), 31.1 ± 5.8, Baseline BMI: 28.9 ± 7.2 Follow‐up BMI: 29.72 ± 6.8	Baseline: 195.08 ± 66.55 After 2.5 ± 0.9 months: 188.96 ± 69.62	No control group	–	–	Peripheral IGF‐1 was not significantly changed by clozapine within 3 months of initiation (*p* = .57).
5	Ntouros et al. (2018)	Greece	Serum, ELISA	DSM‐IV‐TR, established diagnosis of SCH and a current relapse state, AP free for at least 15 days prior to admission and had received medication for no longer than 3 days prior to the blood sampling (for 1 or all 3 days).	16 (16/0), 35.56 ± 5.56, 27.04 ± 2.43	239.03 ± 79.82	Healthy male controls	23 (23/0), 27.04 ± 2.91, 24.52 ± 2.08	266.66 ± 59.52	No statistically significant difference was found between SCH patients and healthy controls.
6	Okamoto et al. (2021)	Japan	Serum, IRMA	DSM‐V, chronic SCH patients (duration of illness ≥10 years), had been receiving several AP drugs.	65 (33/32), 49 ± 10, 22 ± 4.9	109 ± 38	Healthy controls matched by age and gender.	20 (8/12), 46 ± 7.4, 23 ± 3.8	120 ± 39	No significant difference in peripheral IGF‐1 levels between SCH patients and healthy controls (*p* = .27).
7	Palomino et al. (2013)	Spain	Plasma, ELISA	DSM‐IV, patients with first psychotic episode who were subsequently diagnosed with SCH and treated with AP.	Baseline: 27 (20/7), 24.7 ± NR, NR 1 month: 21 (NR), NR, NR 6 months: 19 (NR), NR, NR 12 months: 16 (NR), NR, NR	Baseline: 182.42 ± 96.13 1 month: 237.60 ± 122.39 6 months: 193.92 ± 74.41 12 months: 178.02 ± 97.63	Age and gender matched healthy controls with absence of any Axis I disorder.	27 (20/7), 24.7 ± 6.8, NR	171.60 ± 94.49	Significant increase of peripheral IGF‐1 levels in SCH patients after 1 month (*p* = .039), but not at any of the other stages examined. Peripheral IGF‐1 did not change significantly after 1 year of AP treatment.
8	Teja (2018)	India	Serum, ELISA	ICD‐10 DCR, drug naïve or drug free (for a minimum of 4 weeks from oral AP and 8 weeks from depot preparations) cases of first episode SCH.	30 (23/7), 30.07 ± 5.77, 19.59 ± 0.90	128.08 ± 39.97	Healthy (normal) controls matched by age and gender.	30 (23/7), 28.03 ± 1.89, 22.72 ± 1.63	111.65 ± 24.33	No significant difference in the peripheral IGF‐1 levels between the two groups (*p* = .059).
9	Venkatasubramanian et al. (2007)	India	Plasma, enzyme‐amplified immunochemiluminescence	DSM‐IV, SCH patients never treated with any psychotropic medications including AP.	44 (23/21), 33.0 ± 7.7, 19.9 ± 3.3	123.7 ± 50.0	Healthy controls matched for age, gender, anthropometric measures and physical activity.	44 (23/21), 32.5 ± 7.6, 20.8 ± 2.8	159.1 ± 67.9	Peripheral IGF‐1 was significantly lower in SCH patients (*p* = .006).
10	Venkatasubramanian et al. (2010)	India	Serum, enzyme‐amplified immunochemiluminescence	DSM‐IV, SCH patients never treated with any psychotropic medications including AP and could complete 3‐month follow‐up (prescribed AP: risperidone, olanzapine, flupenthixol).	33 (20/13), 33.8 ± 8.2, NR	Baseline: 113.9 ± 44.7 After about 3 months: 141.5 ± 58.8	Healthy controls matched for age, gender, socio‐economic status, anthropometric measures and physical activity.	33 (20/13), 32.2 ± 8.0, NR	175.2 ± 63.0	At baseline, peripheral IGF‐1 was significantly lower in SCH patients than healthy controls (p < .0001). After treatment, peripheral IGF‐1 increased significantly (*p* < .0001).
11	Wu et al. (2008)	Taiwan	Serum, ELISA	DSM‐IV, hospitalized obese patients with SCH treated with clozapine from 0.5 to 10.92 years.	71 (NR), 40.4 ± 7.5, 30.1 ± 3.5	134.7 ± 87.6	Age‐matched normal healthy controls (normal‐weight).	51 (NR), 40.9 ± 8.1, 23.2 ± 1.9	232.8 ± 88.6	Peripheral IGF‐1 significantly lower in SCH patients (*p* < .01).
12	Yesilkaya et al. (2021)	Turkey	Serum, RIA	DSM‐V, patients in remission and treatment‐resistant SCH. Adherence to medication was determined by clinical interviews and by reviewing pharmacy and medical records.	Remitted: 55 (37/18), 36.63 ± 8.50, 22.43 ± 2.37 Treatment‐resistant: 62 (46/16), 33.91 ± 8.56, 22.83 ± 2.05 Combined: 117 (83/34), 35.19 ± 8.60, 22.64 ± 2.21	Remitted: 137.54 ± 40.28 Treatment‐resistant: 165.11 ± 40.95 Combined: 152.15 ± 42.76	Healthy controls matched by gender, age and BMI.	60 (40/20), 33.88 ± 7.99, 22.68 ± 2.52	173.37 ± 38.85	Peripheral IGF‐1 was lower in the remitted patients group compared to both treatment‐resistant and healthy control groups (*p* < .001).

Abbreviations: AP, antipsychotic(s); BMI, body mass index; DSM, Diagnostic and Statistical Manual of Mental Disorders; ELISA, enzyme‐linked immunosorbent assay; F, female; ICD, International Statistical Classification of Diseases and Related Health Problems; IRMA, immunoradiometric assay; M, male; *n*, number; NR, not reported; *p*, statistical significance; RIA, radioimmunoassay; SCH, schizophrenia; SCID I, the structured clinical interview for DSM‐IV axis 1 disorders; SD, standard deviation.

### 3.3 Methodological quality (risk of bias) assessment

Methodological quality assessment of individual studies is shown in Table 3. The score of comparative studies ranged from 16 to 20 of 24, while the score of the only non‐comparative study was 13 of 16.

**TABLE 3:** Methodological quality assessment of included studies

	Study	(1)	(2)	(3)	(4)	(5)	(6)	(7)	(8)	(9)	(10)	(11)	(12)	Total score
1	Chao et al. (2020)	2	0	2	2	0	2	2	0	2	2	2	2	18/24
2	Chen et al. (2021)	2	0	2	1	0	2	1	0	2	2	2	2	16/24
3	Demirel et al. (2014)	2	0	2	2	0	2	2	0	2	2	2	2	18/24
4	Howes et al. (2004)	2	2	2	2	0	2	2	1	N/A	N/A	N/A	N/A	13/16
5	Ntouros et al. (2018)	2	0	2	2	0	2	2	0	2	2	1	2	17/24
6	Okamoto et al. (2021)	2	0	2	2	0	2	2	0	2	2	2	2	18/24
7	Palomino et al. (2013)	2	2	2	1	0	2	1	0	2	2	2	2	18/24
8	Teja (2018)	2	0	2	2	0	2	2	0	2	2	1	2	17/24
9	Venkatasubramanian et al. (2007)	2	2	2	2	0	2	2	0	2	2	2	2	20/24
10	Venkatasubramanian et al. (2010)	2	0	2	2	0	2	2	0	2	2	2	2	18/24
11	Wu et al. (2008)	2	0	2	2	0	2	2	0	2	2	1	2	17/24
12	Yesilkaya et al. (2021)	2	0	2	2	0	2	2	1	2	2	2	2	19/24

**Abbreviations**: (1) A clearly stated aim; (2) inclusion of consecutive patients; (3) prospective collection of data; (4) endpoints appropriate to the aim of the study; (5) unbiased assessment of the study endpoint; (6) follow‐up period appropriate for the aim of the study; (7) loss to follow up less than 5%; (8) prospective calculation of the study size. Additional criteria for comparative studies: (9) an adequate control group; (10) contemporary groups; (11) baseline equivalence of groups; (12) adequate statistical analyses. N/A, not applicable.

### 3.4 Schizophrenia patients regardless of the antipsychotic treatment status versus controls

**FIGURE 2:**
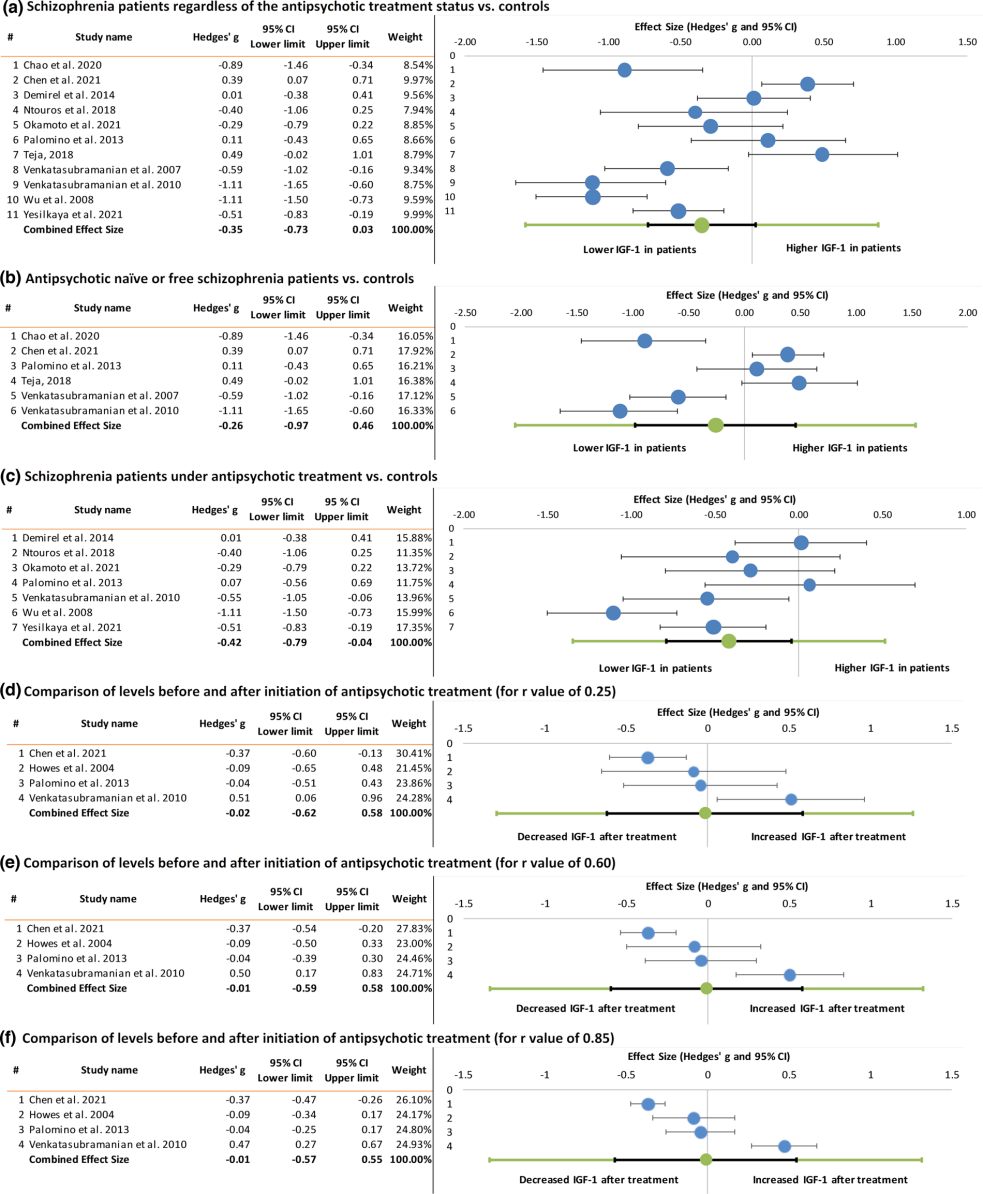
Forest plots showing effect sizes of differences of peripheral insulin‐like growth factor 1 (IGF‐1) levels between schizophrenia patients and controls and changes after antipsychotic treatment

A total of 1018 individuals from 11 studies (Chao et al., 2020; Chen et al., 2021; Demirel et al., 2014; Ntouros et al., 2018; Okamoto et al., 2021; Palomino et al., 2013; Teja, 2018; Venkatasubramanian et al., 2007, 2010; Wu et al., 2008; Yesilkaya et al., 2021) were included in the meta‐analysis (Figure 2a): 596 schizophrenia patients and 422 controls. There was no significant difference in peripheral IGF‐1 levels as 95% CI included zero (Hedges’ g −0.35, 95% CI from −0.73 to 0.03, *Z* = −2.06, *p* = .039). However, between‐study heterogeneity was significant (*Q* = 66.83, *p* < .001, I^2^ = 85.04%). The prediction interval indicates that the next study result will most likely find an effect size between −1.58 and 0.88.

### 3.5 Antipsychotic naïve or free schizophrenia patients versus controls

A total of 495 individuals from six studies (Chao et al., 2020; Chen et al., 2021; Palomino et al., 2013; Teja, 2018; Venkatasubramanian et al., 2007, 2010) were included in the meta‐analysis (Figure 2b): 277 antipsychotic naïve or free schizophrenia patients and 218 controls. No significant difference in peripheral IGF‐1 levels was found (Hedges’ g −0.26, 95% CI from −0.97 to 0.46, *Z* = −0.92, *p* = .359), but between‐study heterogeneity was significant (*Q* = 42.75, *p* < .001, I^2^ = 88.30%). The prediction interval indicates that the next study result will most likely find an effect size between −2.05 and 1.54.

### 3.6 Schizophrenia patients under antipsychotic treatment versus controls

A total of 632 individuals from seven studies (Demirel et al., 2014; Ntouros et al., 2018; Okamoto et al., 2021; Palomino et al., 2013; Venkatasubramanian et al., 2010; Wu et al., 2008; Yesilkaya et al., 2021) were included in the meta‐analysis (Figure 2c): 368 schizophrenia patients under antipsychotic treatment and 264 controls. Schizophrenia patients had significantly lower peripheral IGF‐1 levels compared to healthy controls (Hedges’ g −0.42, 95% CI from −0.79 to −0.04, *Z* = −2.73, *p* = .006), but between‐study heterogeneity was significant (*Q* = 20.26, *p* = .002, I^2^ = 70.38%). The prediction interval indicates that the next study result will most likely find an effect size between −1.35 and 0.52.

### 3.7 Comparison of levels before and after initiation of antipsychotic treatment

A total of 192 schizophrenia patients from four studies (Chen et al., 2021; Howes et al., 2004; Palomino et al., 2013; Venkatasubramanian et al., 2010) were included in the meta‐analysis (Figure 2d–f). There was no significant difference in peripheral IGF‐1 levels before and after initiation of antipsychotic treatment (for *r* value of .25: Hedges’ g −0.02, 95% CI from −0.62 to 0.58, *Z* = −0.09, *p* = .932; for *r* value of .60: Hedges’ g −0.01, 95% CI from −0.59 to 0.58, *Z* = −0.05, *p* = .963; for *r* value of .85: Hedges’ g −0.01, 95% CI from −0.57 to 0.55, *Z* = −0.06, *p* = .953), but between‐study heterogeneity was significant (for *r* value of .25: *Q* = 12.54, *p* = .006, I^2^ = 76.08%; for *r* value of .60: *Q* = 23.07, *p* < .001, I^2^ = 87.00%; for *r* value of .85: *Q* = 57.84, *p* < .001, I^2^ = 94.81%). The prediction interval indicates that the next study result will most likely find an effect size between −1.29 and 1.26 (for *r* value of .25) and −1.34 and 1.32 (for *r* values of .60 and .85).

### 3.8 Subgroup and moderator analyses

The subgroup in which peripheral IGF‐1 levels were measured in plasma (Demirel et al., 2014; Palomino et al., 2013) was without any heterogeneity (I^2^ = 0.00%) in the meta‐analysis where schizophrenia patients were under antipsychotic treatment, but the combined effect size was nonsignificant (Hedges’ g 0.03, 95% CI from −0.02 to 0.08).

In the meta‐analysis of comparison of levels before and after initiation of antipsychotic treatment for all three *r* values, the subgroup of studies conducted in Europe (Howes et al., 2004; Palomino et al., 2013) was without any heterogeneity (I^2^ = 0.00%), while the combined effect size was significant (Hedges’ g −0.06, 95% CI from −0.10 to −0.02) and indicated that peripheral IGF‐1 levels were significantly lower after antipsychotic treatment. Acceptable and nonsignificant level of heterogeneity (I^2^ = 6.11%) was also observed in the subgroup of studies conducted in Europe (Ntouros et al., 2018; Palomino et al., 2013) in the meta‐analysis where schizophrenia patients were under antipsychotic treatment and compared to controls, but the combined effect size was nonsignificant (Hedges’ g −0.16, 95% CI from −0.61 to 0.30).

Results of all remaining moderator and subgroup analyses were not significant.

### 3.9 Sensitivity analysis

Sensitivity analysis has shown that the exclusion of data by two studies would significantly impact the results of the meta‐analysis where schizophrenia patients were included regardless of the antipsychotic treatment status and lead to a significant combined effect size: Chen et al. (2021) (Hedges’ g −0.43, 95% CI from −0.81 to −0.05, *Z* = −2.59, *p* = .010; test for heterogeneity: *Q* = 44.16, *p* < .001, I^2^ = 79.62%; prediction interval from −1.53 to 0.66) and Teja (2018) (Hedges’ g −0.43, 95% CI from −0.80 to −0.06, *Z* = −2.63, *p* = .009; test for heterogeneity: Q = 56.43, p < .001, I^2^ = 84.05%; prediction interval from −1.62 to 0.76).

Exclusion of the data from four studies would lead to a nonsignificant combined effect size in the meta‐analysis where schizophrenia patients were under antipsychotic treatment: Ntouros et al. (2018) (Hedges’ g −0.42, 95% CI from −0.87 to 0.04, *Z* = −2.35, *p* = .019; test for heterogeneity: *Q* = 20.22, *p* = .001, I^2^ = 75.27%; prediction interval from −1.49 to 0.66), Okamoto et al. (2021) (Hedges’ g −0.43, 95% CI from −0.89 to 0.02, *Z* = −2.44, *p* = .015; test for heterogeneity: *Q* = 19.75, *p* < .001, I^2^ = 74.69%; prediction interval from −1.52 to 0.65), Venkatasubramanian et al. (2010) (Hedges’ g −0.39, 95% CI from −0.85 to 0.07, *Z* = −2.20, *p* = .028; test for heterogeneity: *Q* = 20.11, *p* = .001, I^2^ = 75.13%; prediction interval from −1.49 to 0.71), and Yesilkaya et al. (2021) (Hedges’ g −0.39, 95% CI from −0.86 to 0.07, *Z* = −2.17, *p* = .030; test for heterogeneity: *Q* = 20.10, *p* = .001, I^2^ = 75.13%; prediction interval from −1.58 to 0.80).

Sensitivity analysis did not show significant changes with the exclusion of individual studies in the remaining two meta‐analyses (antipsychotic naïve or free schizophrenia patients vs. controls; comparison of levels before and after initiation of antipsychotic treatment).

### 3.10 Publication bias

**FIGURE 3:**
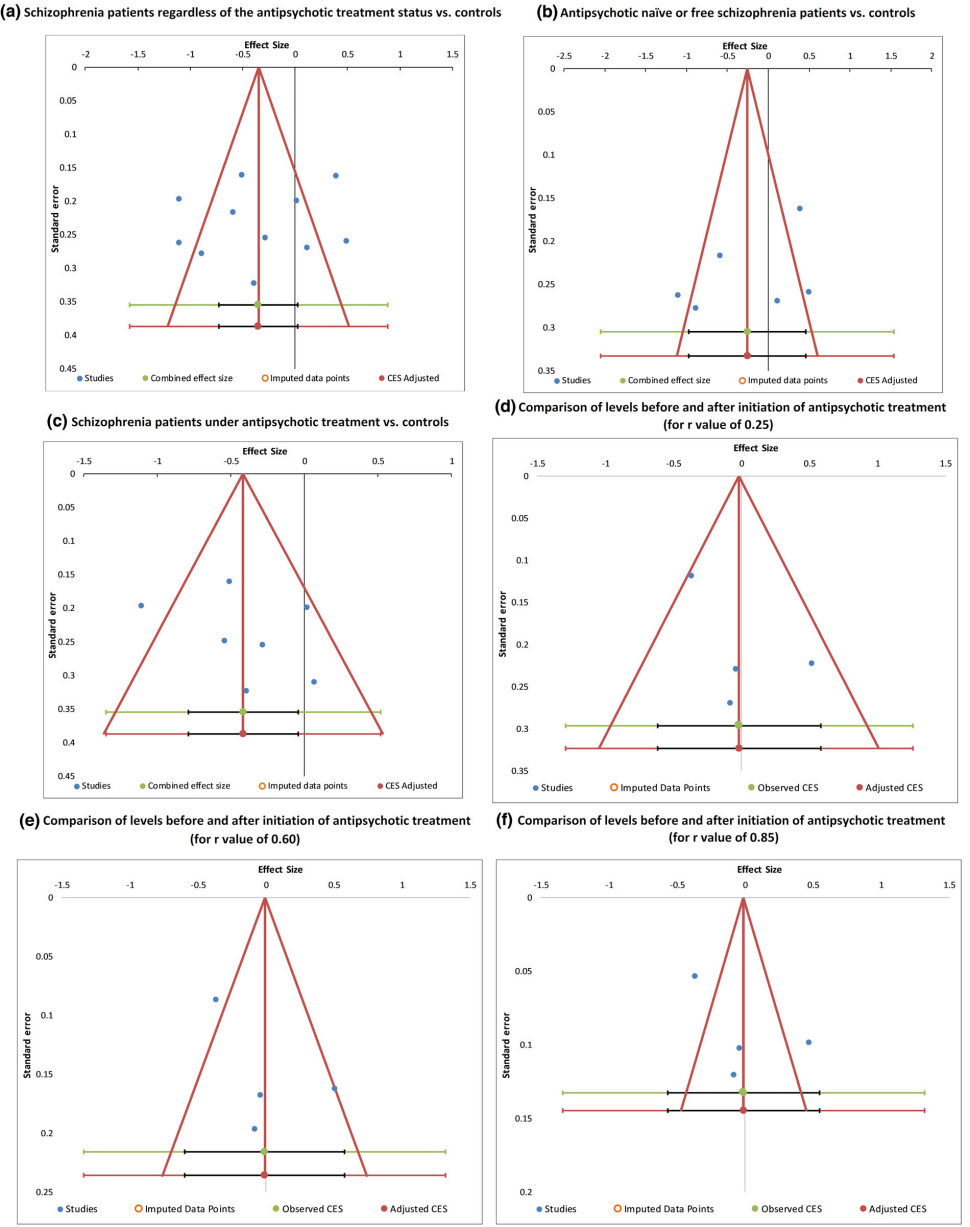
Funnel plots with trim‐and‐fill analysis for differences of peripheral insulin‐like growth factor 1 (IGF‐1) levels between schizophrenia patients and controls and changes after antipsychotic treatment

In all meta‐analyses, the funnel plots indicated that there is no asymmetry in the distribution of effect sizes (see Figure 3: observed and adjusted effect sizes were identical; number of missing studies = 0). Begg and Mazumdar rank correlation test and the Egger regression test also did not detect the presence of significant publication bias (schizophrenia patients regardless of the antipsychotic treatment status vs. controls: *p* = .697 and *p* = .760; antipsychotic naïve or free schizophrenia patients vs. controls: *p* = .188 and *p* = .433; schizophrenia patients under antipsychotic treatment vs. controls: *p* = .293 and *p* = .406; comparison of levels before and after initiation of antipsychotic treatment: for *r* value of .25, *p* = 1.000 and *p* = .503; for *r* value of .60, *p* = 1.000 and *p* = .543; for *r* value of .85, *p* = 1.000 and *p* = .572, respectively).

## 4 DISCUSSIONS

Our meta‐analysis showed that schizophrenia patients under antipsychotic treatment had significantly lower peripheral IGF‐1 levels compared to healthy controls, while no significant difference in peripheral IGF‐1 levels was found between schizophrenia patients regardless of the antipsychotic treatment status and healthy controls, as well as between antipsychotic naïve or free schizophrenia patients and healthy controls, and before and after initiation of antipsychotic treatment. However, high heterogeneity was observed and its potential sources in some of the subgroup analyses included sample type and region, while results were robust only in the sensitivity analysis in the comparison between antipsychotic naïve or free schizophrenia patients versus controls and comparison of levels before and after initiation of antipsychotic treatment.

In the “IGF‐1 deficiency hypothesis” of schizophrenia pathogenesis, it has been previously speculated that low levels of IGF‐1 may increase an individual's susceptibility to schizophrenia possibly by the direct effects of lower IGF‐1 levels on apoptosis, neurogenesis, myelination, synaptogenesis, and dendritic branching during brain development or through reducing the brain's capacity to recover from environmental insults or alternatively by increased apoptosis altering cortical organization through specific effects on the subplate neurons during key periods of neurodevelopment (Gunnell & Holly, 2004). However, our pooled results indicate that there are no significant differences in peripheral IGF‐1 levels between antipsychotic naïve or free schizophrenia patients, as well as between schizophrenia patients regardless of the antipsychotic treatment status and healthy controls. Similarly, single nucleotide polymorphism and variable number tandem repeat polymorphism studies support the conclusion that the IGF‐1 gene is not involved in schizophrenia (Bonvicini et al., 2010; Gunnell et al., 2007). It should also be noted that peripheral IGF‐1 levels may be influenced by a range of factors, such as age, gender, diet, physical activity, BMI, ethnicity, growth hormone, thyroid and parathyroid hormones, cortisol, insulin, estrogens, androgens, inflammatory cytokines, kidney and liver function, diabetic status, and genetic factors (Chanson et al., 2016; Demirel et al., 2014; Frystyk et al., 2010; Gunnell et al., 2007). In addition, a major problem in the clinical application of IGF‐1 measurements is the considerable difference between the results obtained from different assays: For the same sample, different commercial assay kits may produce extremely varied results, with a 2.5‐fold range between the lowest and highest values (Chanson et al., 2016; Pokrajac et al., 2007). Another issue is a lack of agreement on the protocols for collecting and storing samples for measurement of peripheral IGF‐1, which may also impact the measured levels (Frystyk et al., 2010). A well‐known source of error is also the pronounced binding of IGF‐1 to the high‐affinity IGF‐binding proteins (Frystyk et al., 2010).

On the other hand, antipsychotic‐treated schizophrenia patients had significantly lower peripheral IGF‐1 levels compared to healthy controls, but these results were not robust in the sensitivity analysis which may potentially be related to differences in dosage, duration of treatment, or receptor‐binding profiles of different antipsychotics (Siafis et al., 2018; Wu et al., 2008). Also, the subgroup of patients in which peripheral IGF‐1 levels were measured in plasma was without any heterogeneity, but the combined effect size was not significant. It should be noted that the influence of fibrinogen and some coagulation factors cannot be excluded in plasma unlike in serum samples, as well as that sample type was also a source of heterogeneity in the meta‐analysis of peripheral IGF‐1 levels in bipolar disorder and major depressive disorder (Chen et al., 2020).

Antipsychotics may have a complex influence on the growth hormone regulation which ultimately controls the synthesis and secretion of IGF‐1 (Melkersson et al., 2001; Popovic et al., 2007). The secretion of growth hormone is regulated by complex interactions between suprahypothalamic and hypothalamic neurotransmitters and neuropeptides, with dopamine, noradrenaline, and acetylcholine playing important roles (Melkersson et al., 2001). Antipsychotics can block hypothalamic postsynaptic dopamine receptors, so in this way, they may interact with the growth hormone‐regulating systems in hypothalamus, which may result in a decreased hypophyseal growth hormone secretion and consequently reduced production of IGF‐1 (Melkersson et al., 2001). Although antipsychotics mainly block D2 dopamine receptors, they may also block other receptors (e.g., cholinergic and adrenergic) to varying degrees which may also be a contributing factor (Melkersson et al., 2001).

On the other hand, we did not find significant difference in peripheral IGF‐1 levels before and after the initiation of antipsychotic treatment. Conversely, in the subgroup analysis, European patients were found to have significantly lower peripheral IGF‐1 levels after antipsychotic treatment compared to baseline, but peripheral IGF‐1 levels were not significantly different between antipsychotic‐treated schizophrenia patients and healthy controls in this population. However, these results should be interpreted cautiously because a small number of studies (two to four) were included in these analyses. These discrepancies could also be related to differences in used antipsychotics (some patients used monotherapy, while others used combination therapy without specifying which antipsychotic drugs were exactly used), dosage, and duration of treatment. Meta‐analysis of peripheral IGF‐1 levels in bipolar disorder and major depressive disorder also noted that regional differences may be a potential source of heterogeneity, which could be related to different geographical and cultural diet habits (Chen et al., 2020).

There are several limitations of our meta‐analysis that should be mentioned. First, the total number of studies included in some of the analyses was relatively small. Therefore, our results should be interpreted cautiously as some analyses could have been underpowered. Second, marked heterogeneity was observed among the included studies. To exclude the possible confounding effect of food intake on peripheral IGF‐1 levels, we only included studies which specifically stated that fasting samples were used. In addition, we evaluated some of the factors which could be other potential sources of heterogeneity such as age, gender, BMI, sample type, measurement method, region, antipsychotic treatment status, and in some cases duration of antipsychotic treatment, but some of these variables were not available in all included studies, and we also were not able to evaluate effects of other very important factors such as levels of growth hormone, cortisol, insulin and other relevant hormones, relevant binding proteins, and genetic polymorphisms, as well as the influence of individual antipsychotics and their dosage, because these data were either not provided at all in the included studies or were provided but only in a limited number of studies. Third, although we tried to contact the authors of studies which could potentially fulfill our inclusion criteria, not all of them responded and one was not able to provide the relevant data, which also could have affected our results.

## 5 CONCLUSION

In conclusion, schizophrenia patients under antipsychotic treatment seem to have lower peripheral IGF‐1 levels compared to healthy controls. However, additional studies with larger and more homogenous samples are needed to confirm these findings.

## CONFLICT OF INTEREST

The authors declare no conflict of interest.

### PEER REVIEW

The peer review history for this article is available at: https://publons.com/publon/10.1002/brb3.2819.

## Supporting information

Appendix 1. List of excluded publications with reasons

## Data Availability

All data generated or analyzed during this study are included in this published article and its supplementary information files.
