# Supplementary material for: Meta‐analysis of peripheral insulin‐like growth factor 1 levels in schizophrenia
Source: Brain Behav. 2022 Nov 30;13(1):e2819. doi: 10.1002/brb3.2819 (PMC9847627; doi:10.1002/brb3.2819)
Supplement: Supplementary file 1 — Appendix 1. List of excluded publications with reasons [file BRB3-13-e2819-s001.pdf]

Appendix 1. List of excluded publications with reasons for exclusion

	Reference to the publication	Reason for exclusion
1	Melkersson KI, Hulting AL, Rane AJ. Dose requirement and prolactin elevation of antipsychotics in male and female patients with schizophrenia or related psychoses. Br J Clin Pharmacol. 2001;51(4):317-24.	Schizophrenia patients were combined with patients with some other psychiatric diagnosis and no comparison with healthy controls and/or before and after initiation of antipsychotics.
2	Melkersson KI, Hulting AL, Brismar KE. Elevated levels of insulin, leptin, and blood lipids in olanzapine-treated patients with schizophrenia or related psychoses. J Clin Psychiatry. 2000;61(10):742-9.	Schizophrenia patients were combined with patients with some other psychiatric diagnosis and no comparison with healthy controls and/or before and after initiation of antipsychotics.
3	Melkersson KI, Dahl ML. Relationship between levels of insulin or triglycerides and serum concentrations of the atypical antipsychotics clozapine and olanzapine in patients on treatment with therapeutic doses. Psychopharmacology (Berl). 2003;170(2):157-66.	Schizophrenia patients were combined with patients with some other psychiatric diagnosis and no comparison with healthy controls and/or before and after initiation of antipsychotics.
4	Melkersson KI, Hulting AL, Brismar KE. Different influences of classical antipsychotics and clozapine on glucose-insulin homeostasis in patients with schizophrenia or related psychoses. J Clin Psychiatry. 1999;60(11):783-91.	Schizophrenia patients were combined with patients with some other psychiatric diagnosis and no comparison with healthy controls and/or before and after initiation of antipsychotics.
5	Silva BA, Cassilhas RC, Attux C, Cordeiro Q, Gadelha AL, Telles BA, Bressan RA, Ferreira FN, Rodstein PH, Daltio CS, Tufik S, de Mello MT. A 20-week program of resistance or concurrent exercise improves symptoms of schizophrenia: results of a blind, randomized controlled trial. Braz J Psychiatry. 2015;37(4):271-9.	No comparison with healthy controls and/or before and after initiation of antipsychotics.
6	Wu MK, Wang CK, Bai YM, Huang CY, Lee SD. Outcomes of obese, clozapine-treated inpatients with schizophrenia placed on a six-month diet and physical activity program. Psychiatr Serv. 2007;58(4):544-50.	No comparison with healthy controls and/or before and after initiation of antipsychotics.
7	Petrikis P, Boumba VA, Tzallas AT, Voulgari PV, Archimandriti DT, Skapinakis P, Mavreas V. Elevated levels of Insulin-like Growth Factor-1 (IGF-1) in drug-naïve patients with psychosis. Psychiatry Res. 2016;246:348-352.	Schizophrenia patients were combined with patients with some other psychiatric diagnosis.
8	Poa NR, Edgar PF. Insulin resistance is associated with hypercortisolemia in Polynesian patients treated with antipsychotic medication. Diabetes Care. 2007;30(6):1425-9.	Schizophrenia patients were combined with patients with some other psychiatric diagnosis.

9	Karanikas E, Manganaris S, Ntouros E, Floros G, Antoniadis D, Garyfallos G. Cytokines, cortisol and IGF-1 in first episode psychosis and ultra high risk males. Evidence for TNF-α, IFN-γ, TNF-β, IL-4 deviation. Asian J Psychiatr. 2017;26:99-103.	Schizophrenia patients were combined with patients with some other psychiatric diagnosis.
10	Tremblay CS. Insulin-like growth factor-I and memory functioning in recent-onset and chronic schizophrenia. Doctoral dissertation. The Wright Institute. 2013.	Schizophrenia patients were combined with patients with some other psychiatric diagnosis and it was not specified if IGF-1 levels were measured after fasting.
11	Huizer K, Van Beveren N, Roder CH, De Jong FH, Fekkes D. Serum IGF-1 is decreased in recent onset schizophrenia patients. Schizophr Res. 2006;81(Supplement):208.	Not specified if IGF-1 levels were measured after fasting.
12	Akanji AO, Ohaeri JU, Al-Shammri SA, Fatania HR. Associations of blood levels of insulin-like growth factor (IGF)-I, IGF-II and IGF binding protein (IGFBP)-3 in schizophrenic Arab subjects. Clin Chem Lab Med. 2007;45(9):1229–31.	No relevant values for effect size calculation and not specified if IGF-1 levels were measured after fasting.
13	Huizer K, van Beveren NJM, Roder CH, Janssen JAM, de Jong FH, Fekkes D. P.3.b.010 Insulin-like growth factor 1 and recent onset schizophrenia. Eur Neuropsychopharmacol. 2007;17:S423.	No relevant values for effect size calculation and not specified if IGF-1 levels were measured after fasting.
14	Lee BH, Kim YK. P.3.c.024 Differences of plasma BDNF, β-NGF, and IGF-1 between responder and non-responder in schizophrenic patients. Eur Neuropsychopharmacol. 2007;17:S437–8.	No relevant values for effect size calculation and not specified if IGF-1 levels were measured after fasting.
15	Yesilkaya UH, Gica S, Ilnem MC, Sen M, Ipekcioglu D. Evaluation of IGF-1 as a novel theranostic biomarker for schizophrenia. Eur Neuropsychopharmacol. 2021;53:S328-S329.	Conference abstract reporting data from an already included study.
16	Teja VSK, Das B, Mehta VS. Insulin-like growth factor-1 in first-episode schizophrenia: A cross-sectional study. Indian J Psychiatry. 2018;60(5):88.	Conference abstract reporting data from an already included study.
17	Kütükcü A. Atipik antipsikotik ilaç kullanan kronik şizofreni hastalarının metabolik profilleri. Düşük IGF-I düzeylerinin şizofreni ve metabolik sendrom ile ilişkisi [Metabolic profiles of chronic schizophrenia patients taking atypical antipsychotics. Correlations of low levels of IGF-I with schizophrenia and metabolic syndrome]. Doctoral Thesis. Instabul, Turkey: İstanbul Üniversitesi, Cerrahpaşa Tıp Fakültesi, Psikiyatri Anabilim Dalı; 2010.	Dissertation reporting data from an already included study.
